# A Novel Ex Vivo Method for Visualizing Live-Cell Calcium Response Behavior in Intact Human Tumors

**DOI:** 10.1371/journal.pone.0161134

**Published:** 2016-08-18

**Authors:** James Koh, Joyce A. Hogue, Julie A. Sosa

**Affiliations:** 1 Department of Surgery, Duke University Medical Center, Durham, North Carolina, United States of America; 2 Duke Cancer Institute and Duke Clinical Research Institute, Duke University Medical Center, Durham, North Carolina, United States of America; Universita degli Studi di Bari Aldo Moro, ITALY

## Abstract

The functional impact of intratumoral heterogeneity has been difficult to assess in the absence of a means to interrogate dynamic, live-cell biochemical events in the native tissue context of a human tumor. Conventional histological methods can reveal morphology and static biomarker expression patterns but do not provide a means to probe and evaluate tumor functional behavior and live-cell responsiveness to experimentally controlled stimuli. Here, we describe an approach that couples vibratome-mediated viable tissue sectioning with live-cell confocal microscopy imaging to visualize human parathyroid adenoma tumor cell responsiveness to extracellular calcium challenge. Tumor sections prepared as 300 micron-thick tissue slices retain viability throughout a >24 hour observation period and retain the native architecture of the parental tumor. Live-cell observation of biochemical signaling in response to extracellular calcium challenge in the intact tissue slices reveals discrete, heterogeneous kinetic waveform categories of calcium agonist reactivity within each tumor. Plotting the proportion of maximally responsive tumor cells as a function of calcium concentration yields a sigmoid dose-response curve with a calculated calcium EC50 value significantly elevated above published reference values for wild-type calcium-sensing receptor (CASR) sensitivity. Subsequent fixation and immunofluorescence analysis of the functionally evaluated tissue specimens allows alignment and mapping of the physical characteristics of individual cells within the tumor to specific calcium response behaviors. Evaluation of the relative abundance of intracellular PTH in tissue slices challenged with variable calcium concentrations demonstrates that production of the hormone can be dynamically manipulated ex vivo. The capability of visualizing live human tumor tissue behavior in response to experimentally controlled conditions opens a wide range of possibilities for personalized ex vivo therapeutic testing. This highly adaptable system provides a unique platform for live-cell ex vivo provocative testing of human tumor responsiveness to a range of physiological agonists or candidate therapeutic compounds.

## Introduction

Primary hyperparathyroidism (PHPT) is a common endocrine neoplastic disorder caused by a disruption of appropriate calcium sensing in parathyroid gland tumors. The morbidity of PHPT can be significant, including bone loss and fracture, nephrolithiasis, cardiovascular and gastrointestinal disease, and neurocognitive impairment [1]. These symptoms arise secondary to a metabolic disturbance in calcium homeostasis imparted by dysregulated parathyroid hormone (PTH) secretion due to a failure of calcium sensing in culprit adenomatous or hyperplastic parathyroid glands [2]. This loss of calcium responsiveness has historically been attributed to silencing of the calcium sensing receptor (CASR), a class C GPCR that is the central component of the biochemical pathway linking extracellular calcium sensing to the regulated secretion of parathyroid hormone (PTH) [3, 4]. Inactivation of CASR coincident with the emergence of parathyroid neoplasia is the presumptive primary mechanism for the loss of calcium sensing in PHPT [5–8]. However, CASR genetic lesions are not found in sporadic PHPT [9–11], and multiple lines of evidence from our laboratory [12, 13] and others [14–18] indicate that tumor aggregate CASR abundance is not predictive of relative calcium responsiveness. Moreover, we have recently shown in dispersed cell studies that parathyroid adenomas are comprised of functionally distinct cellular subtypes that differ in their relative sensitivity to calcium stimulation despite equivalent levels of CASR expression in each population [13, 19]. This evidence of intratumoral heterogeneity in the composition and biochemical behavior of parathyroid adenomas highlights the need for a means to interrogate the intrinsic calcium responsiveness of parathyroid tumors as a direct functional readout of the underlying calcium-sensing deficit in PHPT.

Mechanistic assessment of calcium sensing capacity in parathyroid tumors requires live-cell functional interrogation of biochemical signaling. While dispersed cell approaches have historically proven useful for this purpose, the applicability of these studies to intact tumor behavior is uncertain. To avoid the potential of input bias from selective dispersed cell survival or recovery and to preserve the effects of regional intratumoral heterogeneity, we developed an intact tissue live-cell imaging approach that allows for conditional ex vivo provocative testing of human tumor behavior. In this study, we document individual cellular patterns of calcium responsiveness within biochemically and cytopathologically verified intact parathyroid adenoma tissue and relate these behaviors to parathyroid hormone production and cumulative tumor calcium sensitivity. By linking live-cell readouts of GPCR signaling to ex vivo intact tissue imaging, the novel methodology described here provides a generalizable approach for examining other disease contexts where heterogeneous tumor composition is apparent but no means exists to probe the functional consequences of this heterogeneity in a native, live tissue context.

## Results

**Fig 1:**
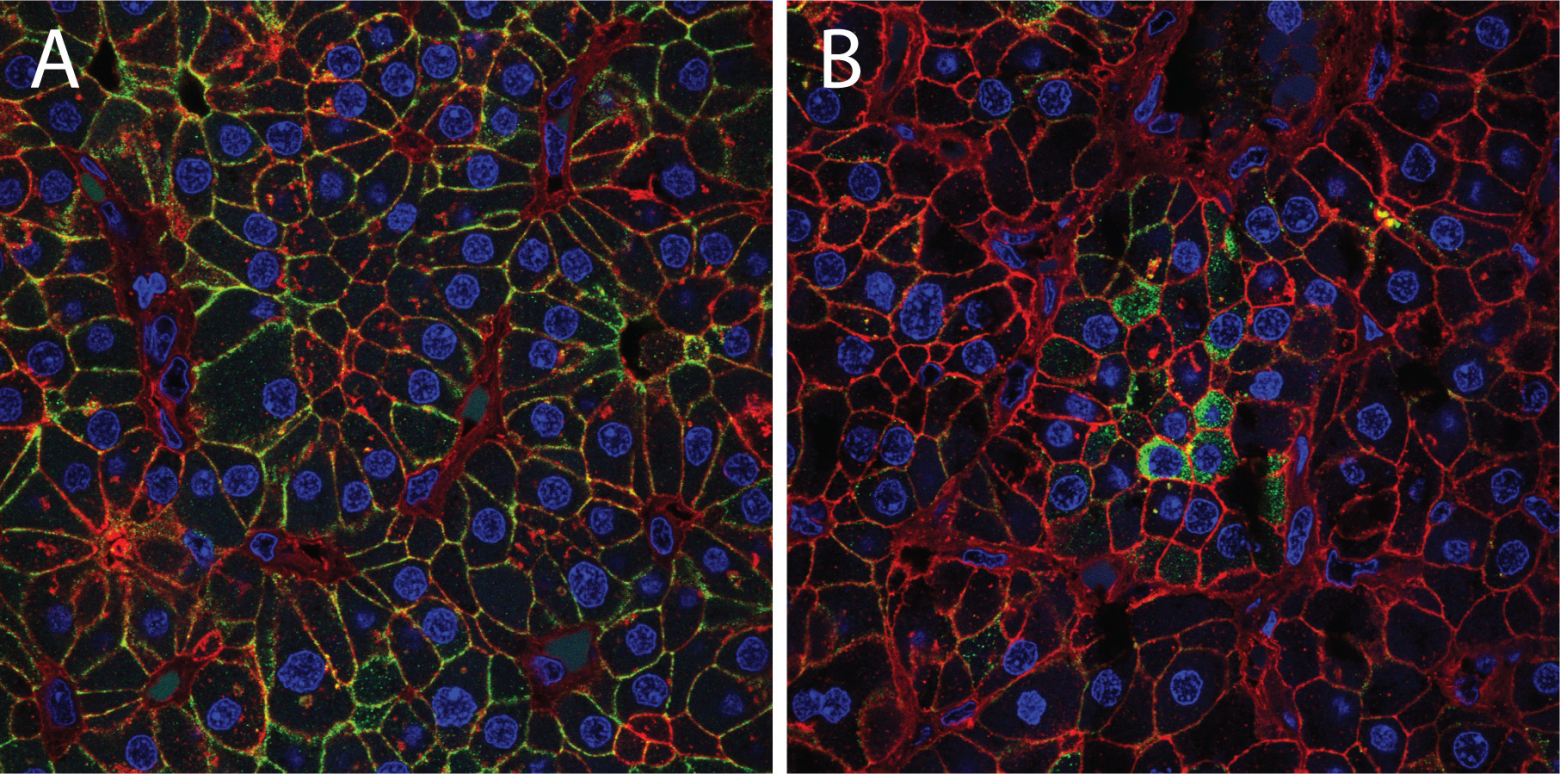
Confocal microscopy image of CASR localization in a primary parathyroid adenoma. (A) region with primarily membrane localized CASR; (B) region with CASR confined to intracellular vesicles in a field of non-expressing cells. Images are 80 micron optical sections. Green = CASR; blue = DAPI; red = WGA, a plasma membrane marker.

We have previously shown that heterogeneous patterns of biochemical responsiveness to calcium stimulation can be found within human parathyroid dispersed cell isolates [13, 19]. To investigate the basis for this functional diversity, we sought to examine the activity and distribution of calcium sensing pathway components in the native context of intact parathyroid tumor tissue. CASR is the central component of the biochemical signaling pathway that regulates cellular responsiveness to changes in extracellular calcium levels [20]. Contextual cues from the physical microenvironment of the intact parathyroid are likely to play a vital role in the subcellular localization and trafficking of CASR, and the disruption of tissue organization by parathyroid tumors may thus compromise native calcium responsiveness. Consistent with this idea, we have frequently observed marked intratumoral regional variations in the subcellular localization of CASR in primary parathyroid adenomas. Different regions of a single tumor can manifest distinct patterns of CASR protein localization, with certain areas displaying the predominantly membrane-bound pattern typical of normal tissue (Fig 1A) co-existing with other areas where CASR is absent or concentrated in cytoplasmic structures (Fig 1B). Patterns such as these can be found in the majority of parathyroid tumors we have examined to date (S1 Fig). Because continual insertion of CASR into the plasma membrane is required for maintenance of calcium sensitivity, regional aberrant localization patterns suggest disruption of CASR protein movement in a subset of the tumor as a mechanism for the loss of calcium sensing in PHPT.

**Fig 2:**
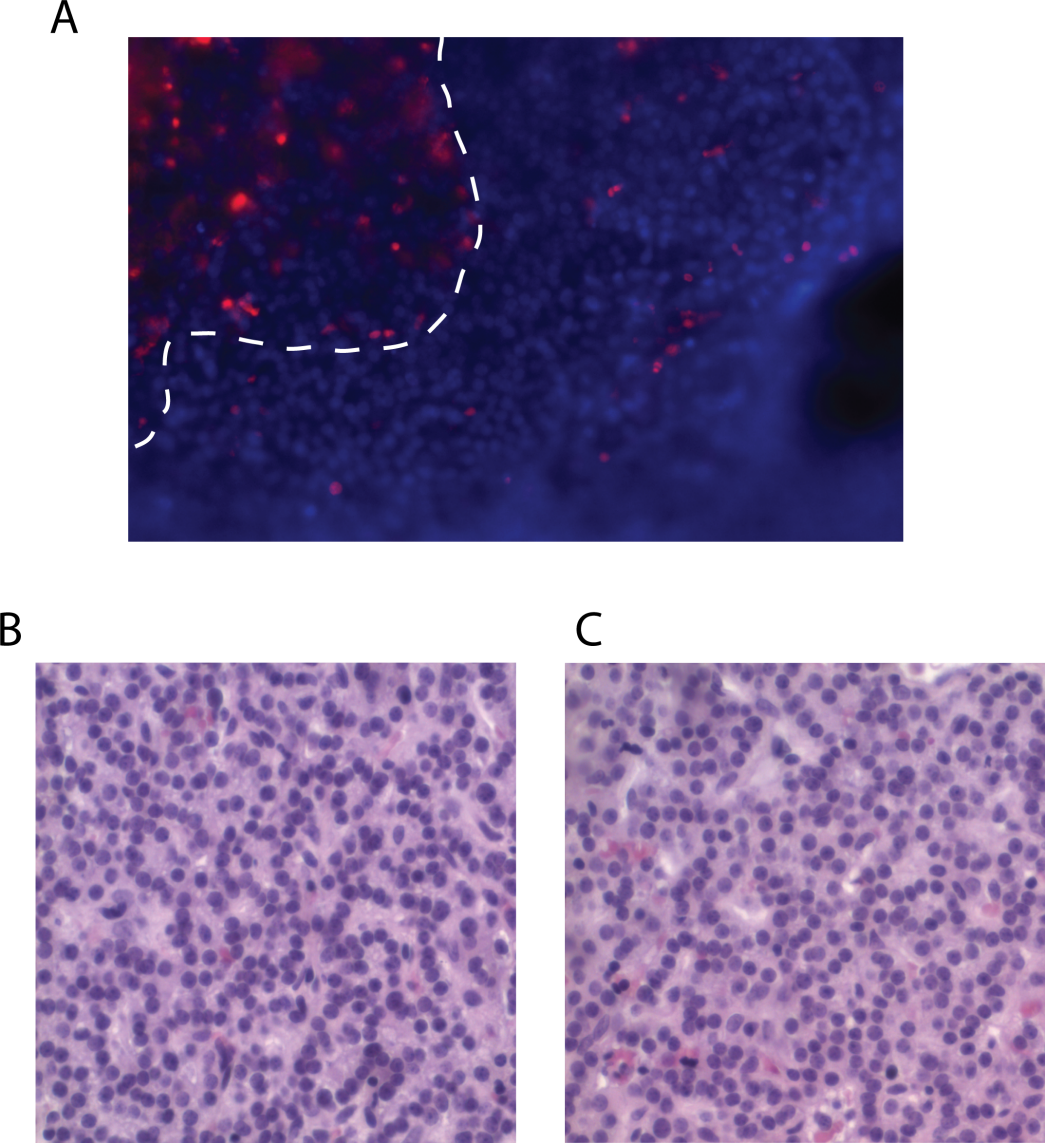
Live-cell vibratome sections of human parathyroid tumor tissue. (A) Parathyroid adenoma slice culture viability. Section is stained with the propidium iodide (red) and Hoechst 33342 (blue). White dashed line indicates the plateau boundary of the vibratome cut surface fracture plane. (B) Hematoxylin/eosin stained sections of a primary parathyroid adenoma and (C) a slice culture specimen derived from the same tumor after 7 days in culture (right panel). Magnification is 200X.

**Fig 3:**
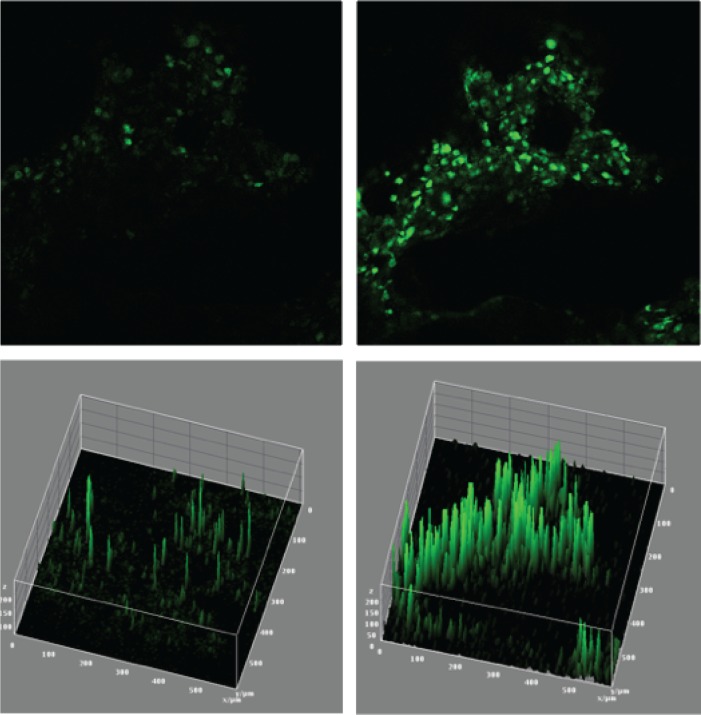
Ionomycin-induced flux response in a viable human parathyroid tumor section. Images are z-stack projections captured with a 20X immersion confocal objective. Images of the same field taken prior (A, C) or 60 seconds after ionomycin addition (B, D). Upper panels are single frame fluorescence emission images; lower panels are three dimensional histogram plots of the same image fields. Blue = Hoechst 33342. Green = activated Fluo4-AM.

While documentation of CASR localization in fixed parathyroid tumor sections provides important information on protein abundance and subcellular compartmentalization, static immunohistochemical analysis does not provide direct insight into functional activity and dynamic responsiveness to extracellular calcium challenge. To address this need, we prepared viable tumor tissue for functional interrogation of calcium responsiveness utilizing the intracellular calcium flux indicator Fluo-4-AM. Parathyroid tumor tissue vibratome-sectioned to 300-micron slice thickness retained excellent viability throughout the live-cell observation period (Fig 2). Dead cells identified by permeability to propidium iodide (red cells) were only transiently detectable and were largely confined to the fracture plane of the vibratome blade (plateau boundary demarcated by the white dotted line)(Fig 2A). The adenoma section (Fig 2B) retained the native tissue architecture of the parental tumor (Fig 2C). To test whether the thick sections could be effectively loaded with the cell-permeant Fluo-4-AM indicator, we incubated viable tumor tissue slices in Fluo-4-AM loading buffer for two hours and then stimulated the specimens with 10 micromolar ionomycin, an agent that provokes uniform release of intracellular calcium stores. Fluorescent intensity in the Fluo-4-AM emission channel was then monitored over a 5-minute observation period. As shown in Fig 3, calcium flux response can be readily visualized throughout the test section under these conditions. The top two panels are single frame images of fluorescent intensity prior (Fig 3A) and 2 minutes after (Fig 3B) ionomycin treatment. The lower panels are histogram plots of individual cell intensities in the same two image fields (Fig 3C, prestimulation; Fig 3D, ionomycin), with the x/y dimensions in microns corresponding to the areal plane of the image field and the z-axis dimension representing fluorescent intensity. Complete raw image stacks from this experiment are provided as time-ordered *lsm* files in the supplementary data (S2 Fig).

**Fig 4:**
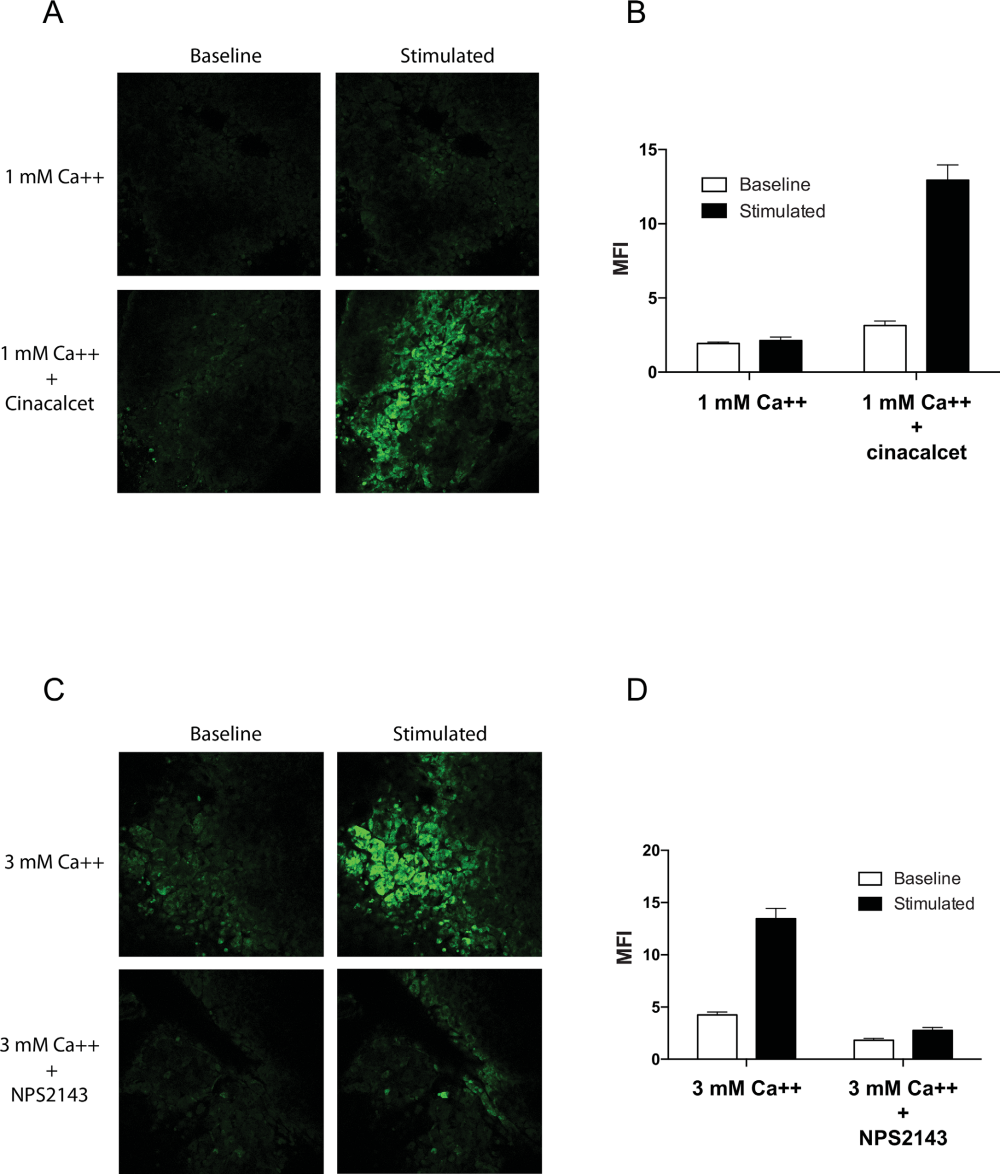
Intracellular flux response to extracellular calcium stimulation is CASR-dependent. Slice culture specimens were challenged with extracellular calcium in the presence of the CASR-specific calcimimetic cinacalcet or the CASR-specific calcilytic agent NPS2143. (A) Single frame immersion confocal images of Fluo-4AM fluorescent intensity at time 0 (“Baseline”) or 1 minute after calcium addition (“Stimulated”), in the presence of 1 mM calcium stimulus alone or in the presence of 1 mM calcium plus 2 micromolar cinacalcet. (B) Quantitation of the image data in (A). Mean fluorescent intensity (MFI) with standard deviation error bars for the cells in each field are plotted for the four conditions shown. Intensity values were captured at the same time points shown in (A). (C) Single frame images of fluorescence intensity before (“Baseline”) and 1 minute after stimulation (“Stimulated”) with 3 mM calcium in the presence or absence of the CASR inhibitory agent NPS2143 at a concentration of 300 nM. (D) Quantitation of the image fields shown in (C).

We next examined the responsiveness of parathyroid adenoma tissue sections to extracellular calcium challenge. The addition of extracellular calcium to calcium-free buffers has under certain circumstances been shown to cause a transient increase in intracellular calcium concentrations through a non-specific process known as the “calcium reintroduction redux” [21]. In order to rule out non-specific influx as a potential confounding factor for our calcium sensing assay, we compared the flux responses of parathyroid tumor sections to extracellular calcium challenge in the presence or absence of the CASR-specific pharmacological compounds cinacalcet, a calcimimetic agent, or NPS-2143, a calcilytic agent [22, 23]. Exposure to 1 mM calcium alone provoked a minimal flux response (1.1 X over baseline), but the same concentration of calcium in the presence of 2 micromolar cinacalcet induced a 4.1-fold increase in intracellular flux (Fig 4A). The mean fluorescence intensities of the cells in each field are shown graphically in Fig 4B. These data demonstrate that the intracellular flux responses observed in our system can be potentiated by the CASR-specific calcimimetic agent cinacalcet. Similarly, at 300 nM the calcilytic agent NPS-2143, a potent CASR inhibitor, strongly blunted the intracellular flux response induced by 3 mM extracellular calcium (Fig 4C and 4D). The stimulatory effect of cinacalcet and the inhibitory effect of NPS-2143 on the intracellular calcium flux provoked by extracellular calcium modulation provides compelling evidence that the flux behaviors we are observing are biochemically specific signaling events mediated through CASR activation.

**Fig 5:**
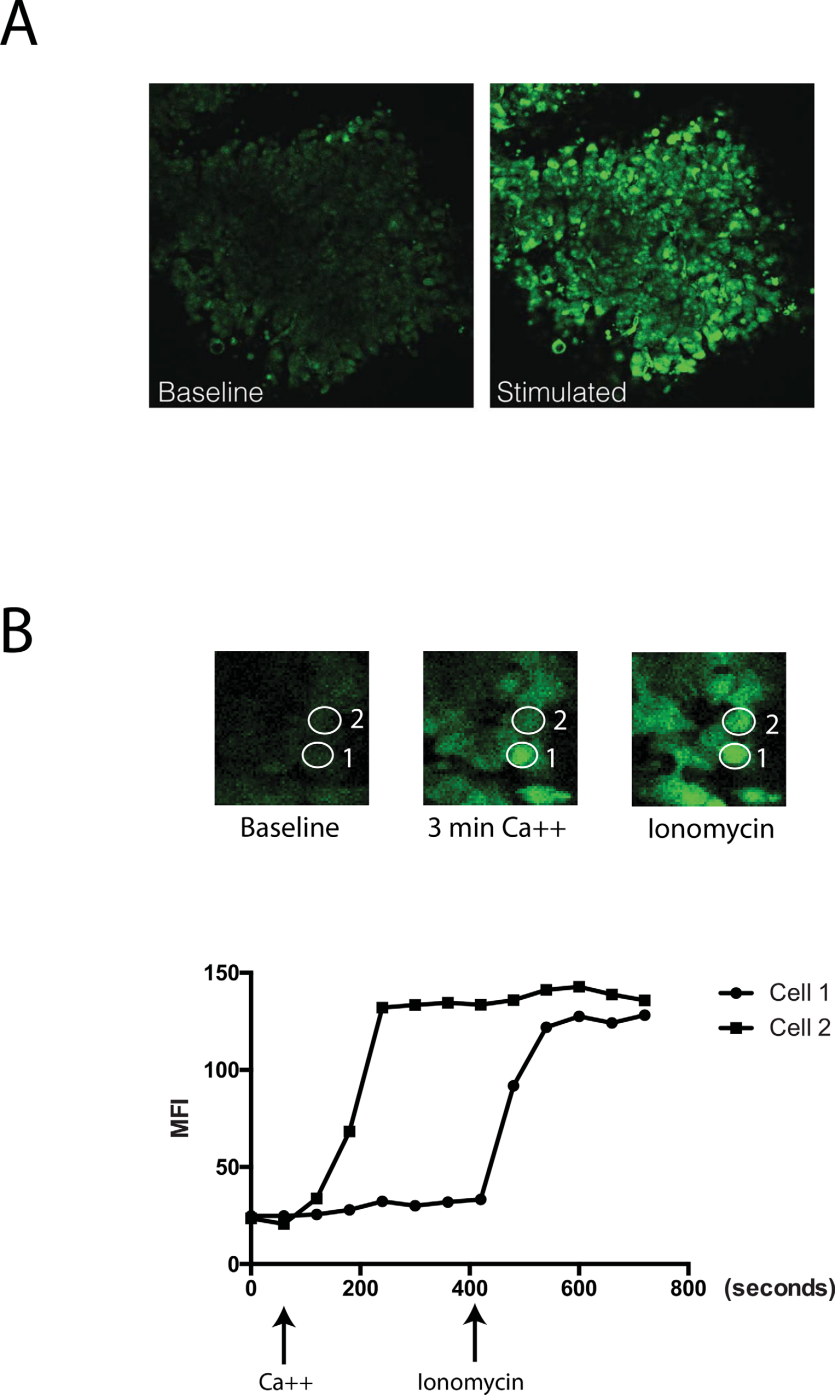
Live-cell flux response to extracellular calcium in an intact human parathyroid tumor section. Images are confocal microscopy z-stack projections from a single field taken at sequential timepoints. (A) Pre-stimulation Fluo-4-AM fluorescence (“Baseline”) and the same field one minute after calcium stimulation (“Stimulated”). (B) Detail view of two individual cells demarcated by numbered ovals captured at three sequential time points: prior to stimulation (“Baseline”), 3 minutes after calcium addition (“Stimulated”), or 2 minutes after ionomycin addition (“Ionomycin”). The accompanying graph plots Fluo4-AM fluorescence mean fluorescence intensity (MFI) over time for the two cells. Green = activated Fluo4-AM. Blue = Hoechst 33342.

Having established the specificity of our functional assay for measuring extracellular calcium sensing behavior, we then examined the responsiveness of parathyroid adenoma sections to calcium challenge. A sequential series of viable tumor sections were prepared from a single-gland parathyroid adenoma and stimulated with a range of extracellular calcium concentrations from 0.5 mM to 10 mM. Exposure to supra-physiological extracellular calcium levels (5 mM) induced a robust and rapid flux response, as visualized by increased fluorescent intensity relative to pre-stimulation baseline (Fig 5A). The majority of cells in this tumor section exhibited a rapid and sustained fluorescent signal consistent with a maximal responder profile [13], while other cells failed to respond under the same conditions. The maximal response profile is defined as sharp (>4X over baseline) increase in induced calcium flux within the first 60 seconds of extracellular calcium stimulus followed by a sustained (<10% intensity deviation) plateau of fluorescence intensity for the duration of the remaining observation period. A detailed view of the fluorescence intensity of two adjacent cells revealed an example of divergent responsiveness to 2 mM calcium stimulation (Fig 5B). Cell 1 exhibited a rapid and sustained flux response to calcium challenge, whereas Cell 2 remained unresponsive under the same conditions (Fig 5B). Subsequent stimulation with ionomycin induced intracellular flux in the calcium non-responsive cell (Cell 2, Fig 5B). A graphical trace of each cell’s mean fluorescence intensity over a 12 minute observation period demonstrated the quantitative difference in their relative responsiveness to extracellular calcium challenge (Fig 5B). Post-flux assay observation of induced fluorescent intensity upon ionomycin treatment confirmed that the cells non-responsive to calcium stimulation retained adequate intracellular calcium stores and contained Fluo-4-AM indicator load comparable to that of the calcium-responsive cells (S3 Fig). A small subset of cells in the tissue sections did not respond to ionomycin, thapsigargin, or A23187, compounds expected to provoke a uniform intracellular flux response [24, 25]. In control experiments, cells non-responsive to these agents were found to stain positively with non-permeant DNA intercalating dye propidium iodide, indicating that these non-responsive cells were non-viable (S4 Fig).

**Fig 6:**
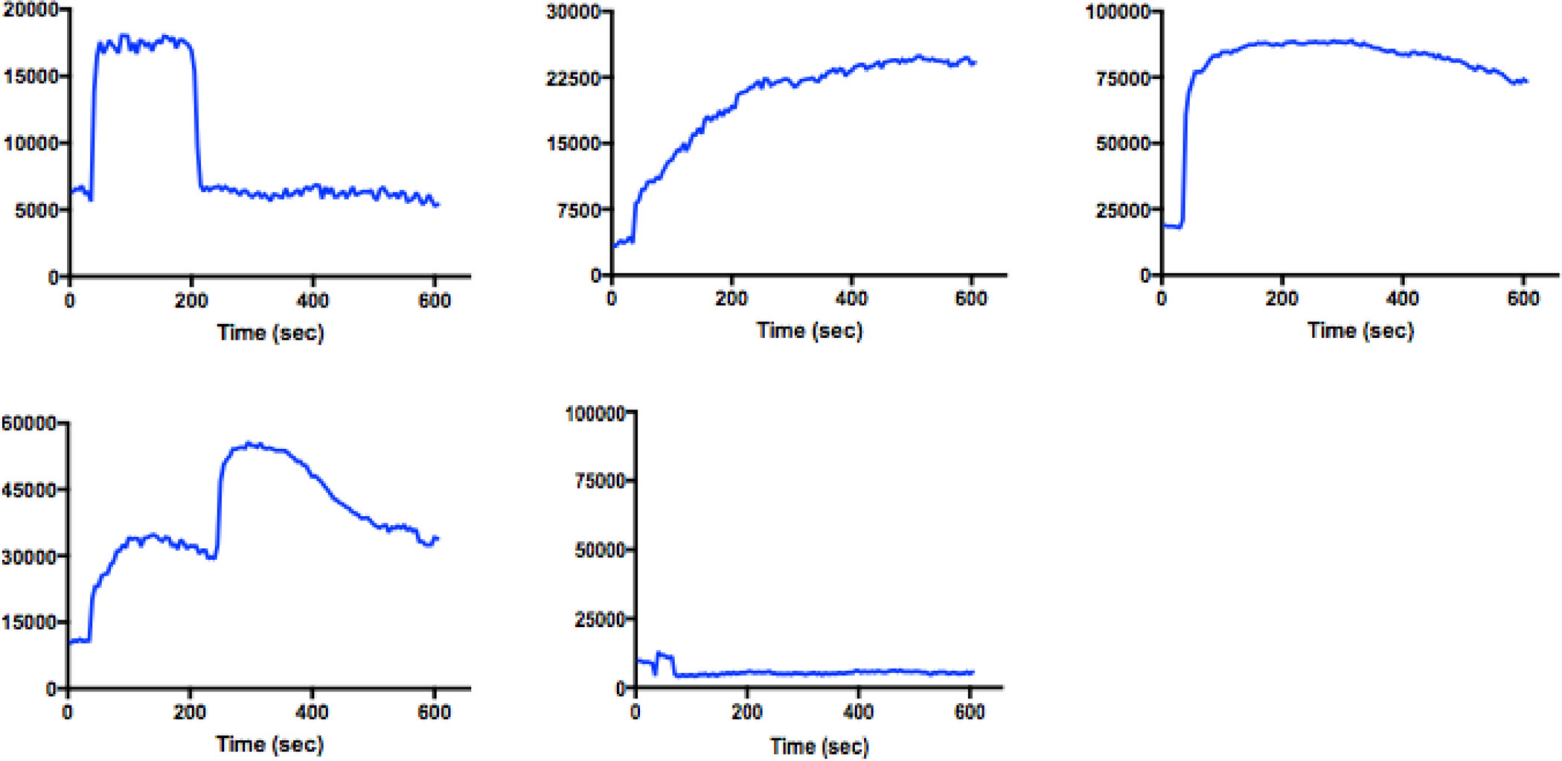
Kinetic profiles of parathyroid tumor cell flux responses. Y-axis = mean fluorescence intensity. X-axis = time in seconds.

The detection of differentially calcium-responsive classes of cells within a parathyroid adenoma was consistent with our earlier studies of parathyroid tumor dispersed cell isolates [13]. Under normocalcemic conditions (1.25 mM Ca++), the range of discrete temporal patterns of calcium flux responses observed within intact tumor tissue was strikingly similar to those we have previously documented [13] in dispersed single cell isolates (Fig 6). As observed with dispersed cells, the proportion of cells exhibiting an immediate and sustained calcium flux response appeared to increase in a calcium dose-dependent manner.

**Fig 7:**
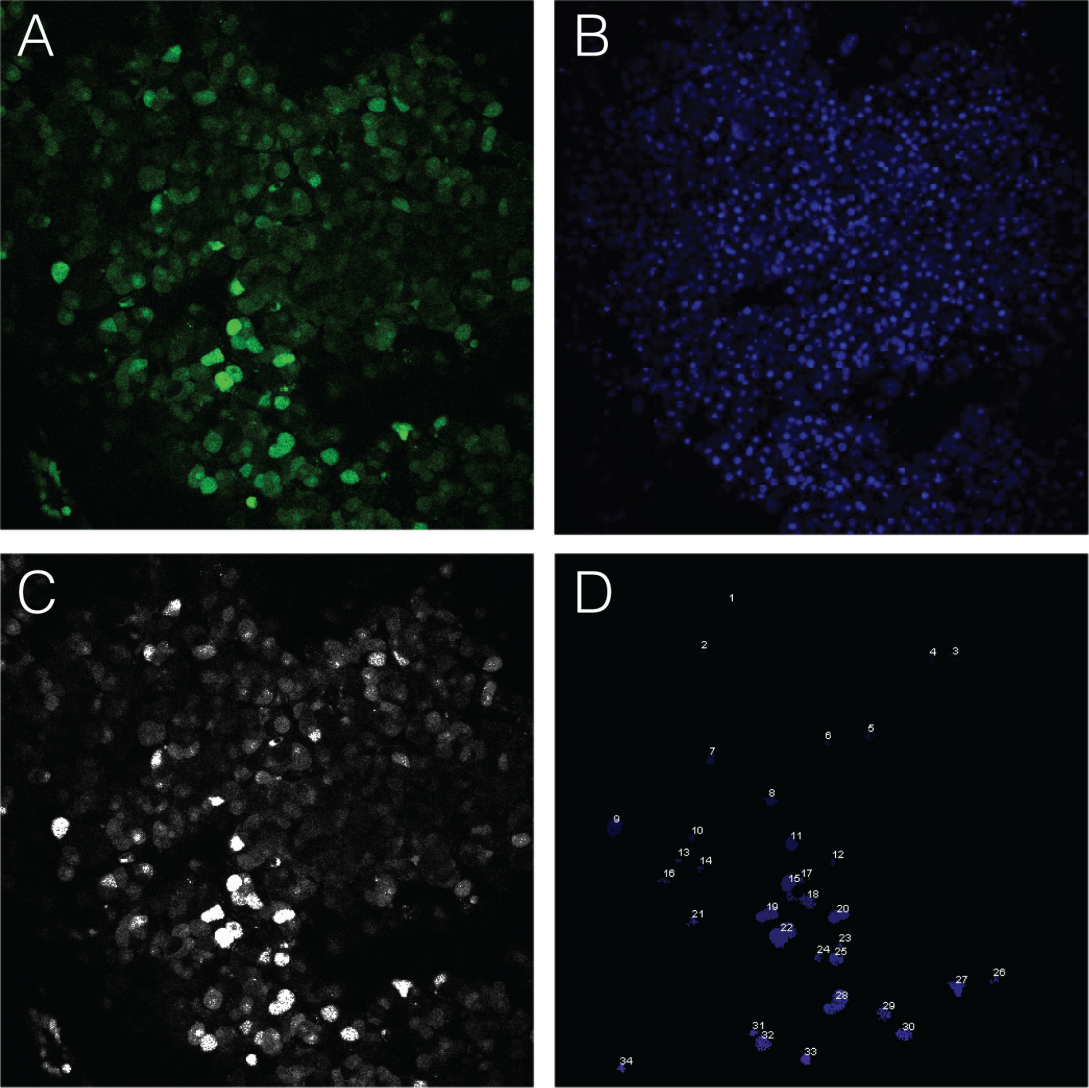
Quantitation of parathyroid adenoma responsiveness to calcium challenge. Fluo-4-AM fluorescence in a tumor section stimulated with 2 mM calcium (upper left panel) was captured at 2 minutes post-treatment and converted to an 8-bit monochrome image for quantitation (upper right panel). Maximal responders, defined as cells exhibiting an immediate onset and sustained plateau of intracellular calcium release greater than 3X over pre-stimulation baseline, are identified as the numbered objects in the lower right panel. The total number of cells in the planar field of view is derived by counting the number of nuclei in the corresponding Hoechst emission channel (lower left panel).

**Fig 8:**
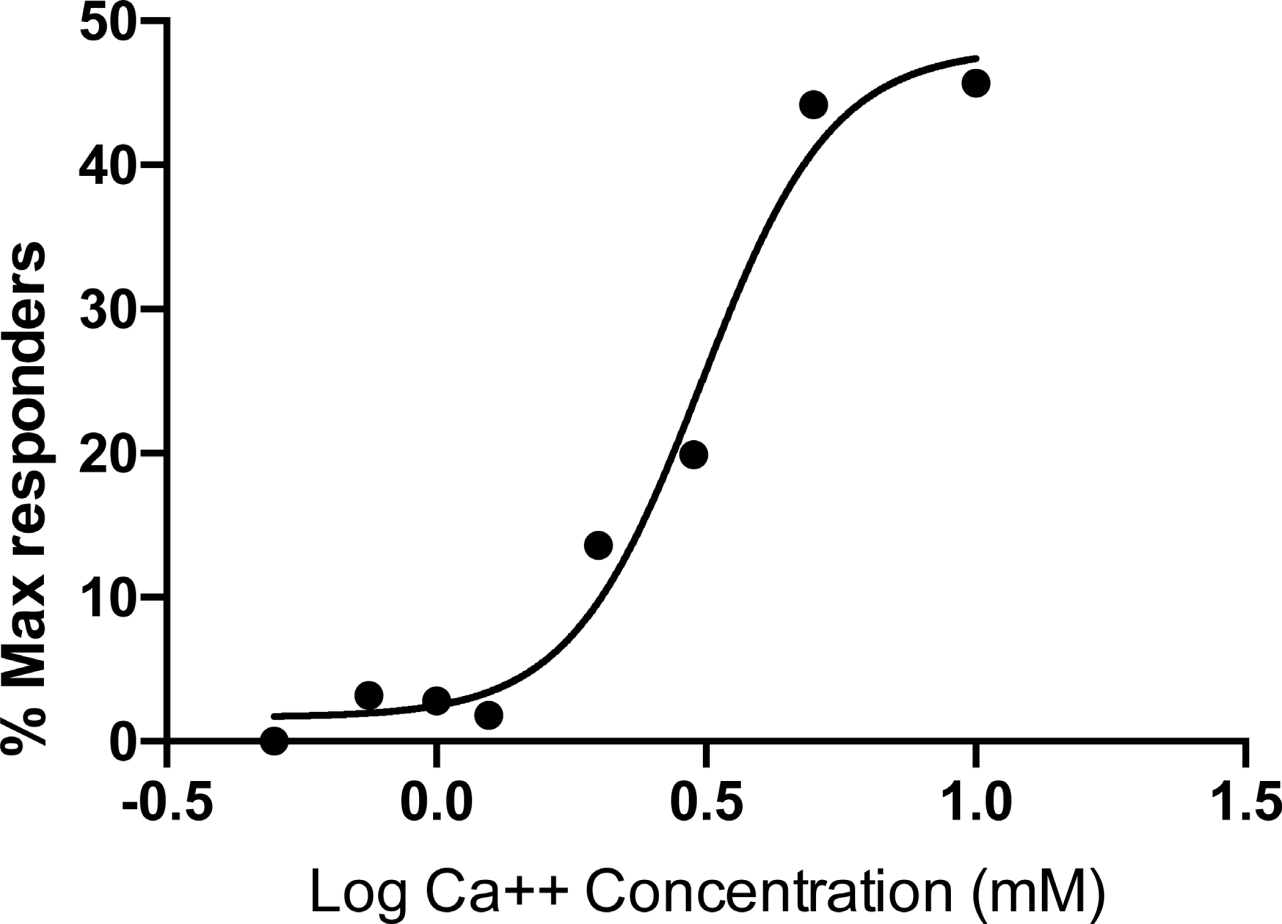
Calcium response setpoint curve. The proportion of cells exhibiting a maximal flux response profile is plotted as a function of log (calcium concentration). Data points are calculated from fields of at least 600 cells at each calcium concentration level.

To determine the relationship between maximally responsive cell behavior and tumor sensitivity to calcium stimulus, we determined the proportion of cells exhibiting an immediate and sustained calcium flux profile in tumor sections challenged with a range of calcium concentrations. Total cell number in each planar image field was determined by counting Hoechst-stained nuclei using the Cell Counter module of ImageJ, and then maximal responders were identified by plotting individual fluorescent intensities over the 9.5-minute post-calcium addition observation period (S5–S13 Figs). A representative example of a tumor section stimulated with 2 mM calcium is shown in Fig 7. A fluorescent signal time frame taken 2 minutes post-stimulation (Fig 7A) is shown with the corresponding nuclear stain for the same time frame and field of view (Fig 7B). Maximal responder cells identified after conversion of the fluorescent image to 8-bit monochrome prior to pixel intensity quantitation are depicted as white regions (Fig 7C). Cells exhibiting the maximally responsive kinetic profile were then counted and identified as numbered objects (Fig 7D). A plot of the proportion of maximally responsive cells as a function of log calcium concentration revealed a sigmoid curve relationship fitted using a standard four-parameter regression model (Fig 8) to reveal a calcium EC50 of 3.20 mM. Because our experimental system is designed to isolate the effects of calcium on CASR activity in the absence of other potential physiological modulators such as magnesium, aromatic amino acids, or spermine, this extrapolated calcium EC50 value may be somewhat higher than what would be observed in vivo. However, by comparison to prior cell culture-based CASR signaling studies using buffers similar to those used in our study, the calcium sensitivity of the parathyroid tumor tissue tested here is clearly well below published measures of wild type CASR reactivity determined in vitro [17]. This result was consistent with the parental tumor conferring attenuated responsiveness to extracellular calcium.

**Fig 9:**
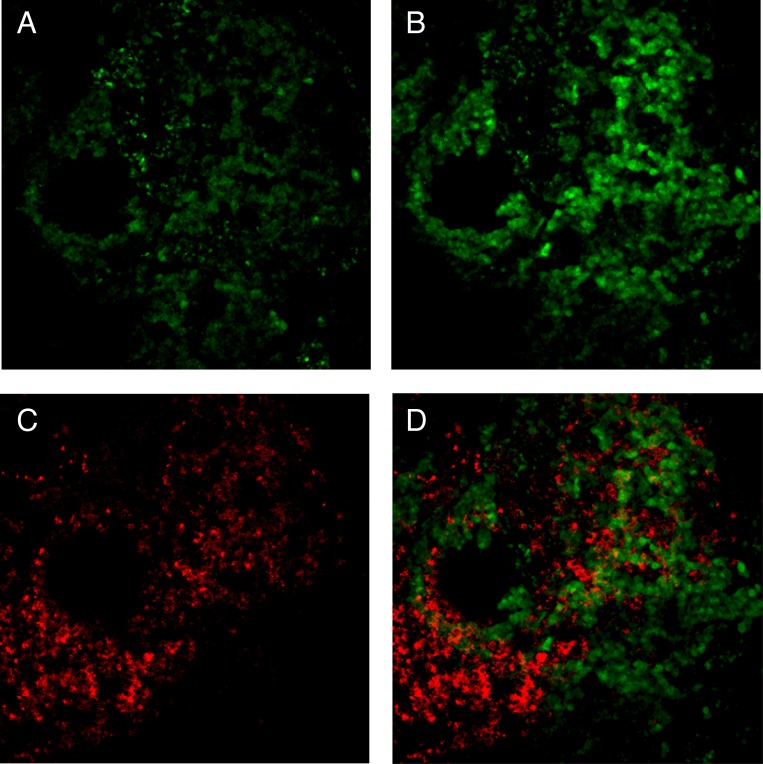
Alignment of live-cell calcium response behavior with PTH abundance in a parathyroid adenoma. (A) Prestimulation baseline flux activity at time 0; (B) same field of view at 2 minutes after stimulation with 2 mM calcium; (C) same field after fixation and immunofluorescent staining for PTH (red); (D) image overlay of (B) and (C). Flux images (A, B) are single timepoint frames from the same time-ordered image stack. Anti-PTH reactivity is visualized by an AlexaFluor555 secondary antibody. Magnification = 200X.

Because positional context is retained in the tissue slice specimens during the calcium response assays, the individual records of each cell’s functional behavior can be aligned with subsequent immunofluorescent imaging of the tissue slice after fixation. To examine the relationship between PTH production and calcium responsiveness in individual parathyroid tumor cells, we probed functionally interrogated tissue sections for PTH abundance. Nuclear staining patterns from live-cell specimens and the same tissue slices imaged after fixation and immunofluorescence staining were employed as landmarks using the registration tool in ImageJ to generate precise alignment of corresponding fields of view in each image set. Fluorescence intensity in the PTH channel was then quantitated and related to live-cell flux response activity. As shown in Fig 9, PTH was largely not coincident with calcium responsiveness. In the example shown, conditionally responsive flux-positive cells were identified by increased green fluorescence at 2 minutes post-stimulation (Fig 9B) relative to the pre-stimulation baseline state (Fig 9A). In this same field, PTH was detected in scattered cells (Fig 9C) that did not appear to align with cells displaying calcium responsive flux activity (Fig 9D). A co-localization score for PTH and flux-positive cells was calculated using the Costes thresholding method embedded in the Coloc2 module of ImageJ. The Pearson’s correlation coefficient R value for co-localization in the images in the 2 mM condition shown in Fig 9 was 0.20, suggestive of a low degree of overlap between the capacity to sense calcium stimulus and the presence of intracellular PTH.

**Fig 10:**
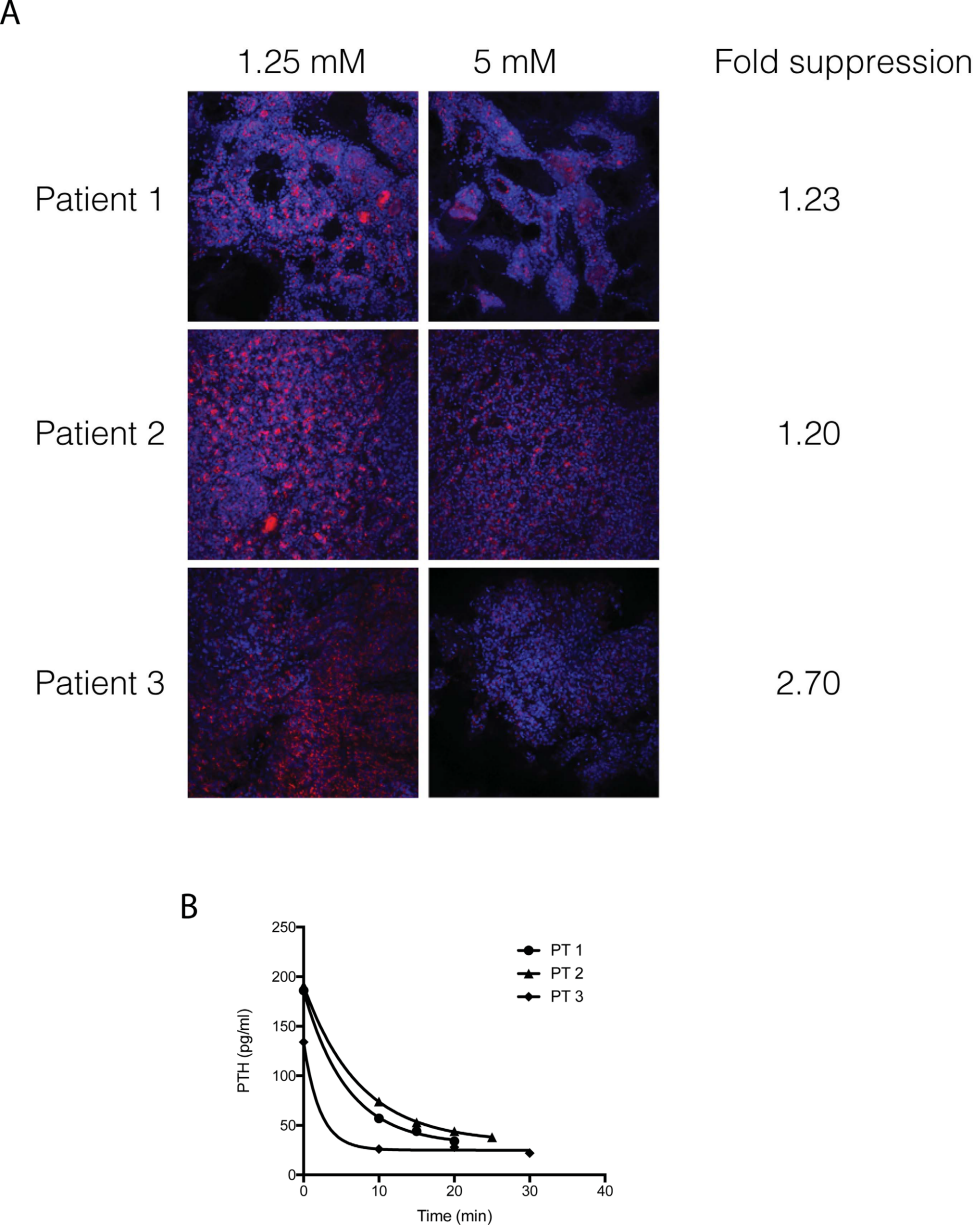
Calcium-sensitive modulation of PTH abundance in live parathyroid adenoma tissue. (A) Tumor sections from three different parathyroid adenoma specimens were challenged by exposure to elevated extracellular calcium (5 mM, right column) and compared to matched sections maintained under normocalcemic conditions (1.25 mM, left column). PTH reactivity is shown in red; nuclei are shown in blue. Fold suppression is calculated as the relative change in mean fluorescence intensity of the anti-PTH signal (AlexaFluor555-conjugated secondary antibody) normalized to baseline fluorescence under normocalcemic conditions. (B) PTH decrement following parathyroidectomy. Circulating levels of intact PTH were determined intraoperatively prior to tumor resection (time 0), at 10 minutes post-resection, and at 5 minute intervals thereafter.

Because uncoupling of calcium sensing from PTH production is the causative physiological defect in PHPT, we next determined whether PTH abundance was suppressible in live parathyroid tumor tissue in response to elevated calcium. Serial thick sections of 3 independent parathyroid tumors were cultured overnight under normocalcemic conditions (1.25 mM Ca++) and subsequently were challenged by a 2-hour incubation in media containing either 1.25 mM or 5.0 mM Ca++. The sections were then fixed and processed for immunofluorescent detection of PTH in the intact tissue slice. A stack of 20 confocal plane images through a z-axis depth of 4.8 microns were captured using a 20X immersion lens, with anti-PTH reactivity visualized by an AlexaFluor555-conjugated secondary antibody. Mean fluorescent intensity was calculated and normalized to each field’s nuclear staining (Hoechst 33342) intensity to control for variations in image field depth and specimen planarity. As shown in Fig 10A, PTH abundance was modestly suppressed by high calcium conditions in two of the three tumors (1.23-fold and 1.20-fold decrease), with a third tumor demonstrating a greater degree of responsiveness (2.70-fold reduction in PTH). The degree of suppressibility did not correlate with relative baseline PTH abundance. PTH distribution at baseline was variable but typically found throughout the tumors with no clearly defined regions of concentration or exclusion. These data demonstrate that the capacity to modulate parathyroid hormone abundance in response to increased extracellular calcium could be directly observed ex vivo under experimentally controlled provocative testing conditions. The rate of intraoperative PTH decline in vivo following tumor excision appeared to correspond to relative calcium suppressibility (Fig 10B). Circulating PTH levels in the two patients (PT 1, PT 2) whose tumors demonstrated less sensitivity to calcium suppression declined at a slower rate than in the patient with a more responsive tumor (PT 3). When fitted to a one phase exponential decay curve, circulating PTH half-life in PT 1 and PT 2 was projected to be 4 and 5.1 minutes, respectively. In contrast, the projected PTH half-life in PT 3 was 1.5 minutes. The percent decrement of circulating PTH following tumor resection, an independent biochemical indicator of post-operative normalization that has been shown to be associated with neurocognitive improvement after parathyroidectomy [26], was 16.4% in PT 3 while PT 1 and PT 2 yielded decrements of 18.4% and 39.7%, respectively. The results from the limited sample set examined here could indicate that the extent of calcium-PTH signaling axis failure in parathyroid tumors may vary among PHPT patients, although larger studies will clearly be required before any clinical conclusions can be drawn. This outcome was consistent with our earlier studies indicating distinct categories of calcium EC50 sensitivity among an unselected cohort of PHPT patients [13].

## Discussion

We have described an image-based approach for visualizing the calcium-responsive behavior of live human parathyroid tumor tissue at single cell resolution while preserving native tissue context. Using this system, we sought to determine whether the physiological calcium-sensing deficit in patients with PHPT is caused by an intrinsic global loss of calcium responsiveness within parathyroid tumors as widely believed [2, 3, 27, 28], or whether functional intratumoral heterogeneity in calcium signaling could indicate a more complex underlying disease mechanism. The results of the present study support the concept that heterogeneous populations of cells with variable kinetic profiles of calcium responsiveness can be found within intact parathyroid adenomas. This demonstration of biochemical heterogeneity implies multifactorial molecular mechanisms of calcium-sensing failure rather than uniform suppression of CASR activity as the operative defect in PHPT culprit parathyroid tumors.

Regional differences in the abundance and subcellular localization of CASR within individual parathyroid adenomas suggests that the capacity to respond to extracellular calcium stimulus would be non-uniform among tumor cells. Consistent with this idea, we observed discrete subgroups of tumor cells exhibiting distinct kinetic patterns of live-cell calcium response biochemical signaling within intact parathyroid tumor tissue slices. The temporal and relative amplitude profiles of these kinetic patterns closely resembled those we had previously observed in preparations of isolated human parathyroid tumor dispersed cell suspensions [13], an indication that diversity in calcium signaling behavior is an inherent property of intact parathyroid tumors and not an artifact of cellular isolation. In the tumors examined to date, cells exhibiting a maximally responsive calcium signaling profile were found to be under-represented, leading to an attenuation of overall calcium sensitivity as manifested by an elevated calcium EC50 value relative to historical standards for wild type CASR sensitivity. Moreover, calcium sensing activity and PTH production were detected in partially overlapping but non-identical subsets of cells. These findings revealed an unexpected degree of functional compartmentalization within parathyroid adenomas, with potential implications for targeted medical management approaches to PHPT.

The uncoupling of PTH production from appropriate calcium sensing is the causative defect in PHPT. Two out of three parathyroid adenomas tested displayed minimal suppression of PTH abundance in response to elevated calcium concentrations, while the same conditions provoked a larger, 2.7-fold decrease in a third tumor. These data suggest that not all PHPT cases share the same underlying degree of deficiency in calcium-responsive PTH modulation. Consistent with this idea, the two patients with less-responsive tumors presented with objective indicators of more severe disease; PT 1 was diagnosed with osteoporosis, while PT 2 presented with nephrolithiasis. These data are consistent with the notion that clinical differences in PHPT disease severity may reflect differential degrees of tumor biochemical responsiveness in calcium sensing. While it is currently not possible to identify clinical correlates associated with ex vivo tumor behavior definitively due to the limited size of the sample group to date, the intriguing diversity of parathyroid tumor behavior suggests that aggregate analysis of a larger cohort of specimens may yet reveal distinct clinicopathological subsets of PHPT.

The broader impact of this study arises from the generalizability of our ex vivo approach to examining functional signaling pathways in human tumors. The methodology is readily adaptable for visualizing the dynamic behavior of biopsy-scale human tumor specimens to a variety of agents or provocative testing conditions in an experimentally tractable system that preserves three dimensional native tissue context. Real-time ex vivo evaluation of live-cell functional activity could provide a powerful new tool to augment conventional static histomorphological assessment of human tumor biopsy material. The ability to interrogate the responsiveness of human tumor cells to physiological agonist engagement with subsequent alignment to in situ histochemical characterization provides an important new ex vivo tool for examining the functional consequences of intratumoral heterogeneity. The demonstration that functional mechanistic insight can be derived from real-time analysis of living human tumor biopsy material is an important step towards developing future diagnostic applications where tumor functional behaviors can provide actionable clinical diagnostic information.

## Materials and Methods

### Procurement of patient material

Parathyroid tumor sections were prepared from surgically resected adenoma tissue obtained from patients with PHPT undergoing parathyroidectomy at Duke University Medical Center. Tissue identity was established by ex vivo aspiration of the parathyroid adenoma with confirmation demonstrated by an off-scale (>5,000 pg/ml) result using the rapid intraoperative PTH assay, along with serial peripheral blood monitoring using the Miami criteria [29] of a >50% decline from pre-operative PTH levels within 10 minutes of tumor resection; results were confirmed by post-operative histopathological assessment of the surgical specimen. All procedures on human subjects were reviewed and approved by the Duke University Institutional Review Board (IRB). Patients preoperatively diagnosed with PHPT were recruited by endocrine surgery faculty and enrolled in the study after providing fully informed consent as described under an active IRB-approved protocol maintained by our research group (Pro00046210). Informed consent was documented in written form for all study participants. De-identified parathyroid adenoma surgical specimens were provided to the laboratory for immediate harvest and recovery of viable tumor sections.

### Tissue sectioning

Viable parathyroid tumor sections were prepared using a Leica VT1000S vibratome with blade vibration frequency set to 90 Hz and blade advance speed at dial setting 1.9, which corresponds to approximately 0.07 mm/sec. Tissue temperature and collection media were maintained at 4 degrees C. The collection media is chilled PBS (Gibco Cat. No. 14190) containing 1X antibiotic/antimycotic solution (Gibco Cat. No. 15240–062) in a sterilized buffer tray used solely for viable tissue collection procedures.

Prior to sectioning, a small piece of tissue representing ~10–15% of the total tissue mass is removed for immediate fixation in neutral buffered formalin for subsequent FFPE processing. The remaining viable tissue is trimmed to remove any obvious fat or loose tissue fragments and is then immersed in a 4% w/v low-melt agarose (BioExpress, Cat. No. E-3128) solution. Once the agarose has hardened, the embedded tissue is excised from the agarose mold into a ~5 mm^3^ cube. The cube is blotted dry to remove excess surface liquid and is then affixed to the vibratome sample holder with waterproof glue (Loctite, Ted Pella Cat. No. 10035). Tissue sections are recovered from the buffer tray using a small histology brush and transferred onto PET transwell inserts (3 micron pore size; VWR Cat. No. 10769–194) in parathyroid culture media [13].

### Tissue slice culture and fluorophore loading

The parathyroid tumor tissue slices are incubated overnight in normocalcemic media [13]. The depth of the incubating media is adjusted, such that the tissue slices rest at an air:liquid interface in a humidified 5% CO_2_ tissue culture incubator. Prior to imaging, the tissue slices are removed from the transwell inserts, transferred to 35 mm dishes, and incubated for 2 hours with the cell-permeant intracellular flux indicator Fluo-4-AM in calcium-free buffer composed of 5.333 mM KCl, 0.441 mM KH_2_PO_4_, 4.167 mM NaHCO_3_, 137.93 mM NaCl, 0.338 mM Na_2_HPO_4_, and 5.55 mM D-glucose (HBSS; Gibco Cat. No. 14175) following the manufacturer’s instructions (ThermoFisher, Cat. No. F14201). One hour prior to imaging, Hoechst 33342 (Invitrogen, Cat. No. H21492) is added to the media at a final concentration of 5 micrograms/ml. Cinacalcet (R-568) was provided by Amgen for research purposes through a Research Program Agreement (Agreement Number 200810737) with Duke University. NPS 2143 hydrochloride (Cat. No. sc-361280) was obtained from Santa Cruz Biotechnology, Inc. Thapsigargin (Cat. No. T9033-0.5 mg) and A23187 (Cat. No. C9275-1MG) were obtained from Sigma.

After incubation, the loading media is withdrawn from the 35 mm dishes containing the individual tissue slices. The tissue slice is positioned in the center of the dish and immobilized by overlaying a weighted nylon mesh (250 micron pore size; Genesee Cat. No. 57–107) with a pre-cut opening in the center, leaving the majority of the tissue exposed but with the margins of the tissue held in place by the mesh. Alternatively, tissue slices were placed directly on a 1 cm^2^ piece of the 250 micron pore nylon mesh and then positioned in a thin layer of low melt agarose (4% w/v in water, GeneMate Cat. No. E-3126-25) in a 35 mm dish. In this latter case, 500 microliters of molten low melt agarose solution is aliquoted in each 35 mm dish and the plates are held on a heat block to maintain the agarose in a molten state. Just before use, the plates are removed from the heat block and the agarose is allowed to cool to approximately 37 degrees C. The tissue slice on the 1 cm^2^ mesh piece is then placed in the center of the dish and the agarose is allowed to solidify immediately at room temperature. Fresh Fluo-4-AM solution is then added back into the dish before imaging.

### Live-cell imaging

Live-cell calcium flux response in the tissue sections was observed using a Zeiss 780 multiphoton laser-scanning upright confocal microscope and Zen 2010 software. Images were captured with a 20X water immersion lens (20X/1.0 Water W Plan-Apochromat 421452–9800, WD 1.8 mm). Frame size was 512 x 512 and the line step was set to 1. The imaging speed setting of 7 resulted in a pixel dwell time of 3.15 microseconds and a scan time of 1.94 seconds per image. The pixel averaging number is 2 and the bit depth is 8. The scaled image size is 606.1 x 606.1 microns, with each pixel equal to 1.19 micron^2^ and the zoom set to 0.7. The pinhole and laser power settings are adjusted for each sample based on pre-stimulation background levels, and typically average 5.11 AU and 0.020 mW, respectively. The optical section thickness is 4.8 microns. For each field, two optical sections are imaged with an 11 micron step.

For each section, 132 sequential images are captured at 5 second intervals over an 11 minute observation period. Fluo-4-AM emission was captured using GaAsP high QE 32 channel spectral array detectors and a standard green fluorescence filter cube. In parallel, Hoechst 33342 blue fluorescence was captured from the same fields to visualize nuclei within the imaging plane. The image stack begins with a 30 second baseline period (six consecutive images), after which the calcium stimulus is added. Individual tissue slices from each adenoma were challenged with final concentrations of extracellular calcium (CaCl_2_) at 0.5, 0.75, 1.0, 1.25, 2.0, 3.0, 5.0, or 10.0 mM. At the end of the primary observation period, ionomycin (ThermoFisher, Cat. No. 124222) is added to the media at a final concentration of 10 micromolar, and flux intensity was recorded over an additional one minute period as a positive control to verify adequacy of fluophore loading and intracellular calcium content in the tissue section. Cells failing to demonstrate an ionomycin-induced flux response were excluded from analysis. Stimulation with 10 nM thapsigargin [24] or 5 micromolar A23187 [25] as alternative positive controls revealed a similar degree of loading efficiency and calcium flux response capacity (Supplementary Data S4).

### Immunofluorescence

After imaging, the tissue sections were formalin-fixed using a modified version of a protocol previously described for thick section imaging of neuronal tissue [30]. The fixed sections were permeabilized by incubation in 20% DMSO (Sigma Cat. No. D2650)/2% Triton X-100 (Sigma Cat. No. T8787) in PBS (Gibco Cat. No. 14200) for 2 hours at room temperature on a horizontal shaker. The sections were then blocked overnight in the same solution containing 5% BSA (Sigma Cat. No. A2153). The next day, the tissue slices were transferred into a solution containing an anti-PTH primary antibody (Novus, clone BGN/1F8) or isotype matched mouse IgG, each at a 1:500 dilution in 20% DMSO/2% Triton X-100/2.5% BSA. The tissue slices were each placed in 500 microliters of primary antibody solution in individual heat sealed pouches to prevent evaporation and were incubated for 3 days at 4 degrees with gentle agitation. The slices were subsequently removed from the pouches and washed in four changes of 20% DMSO/2% Triton X-100/2.5% BSA solution over 6 hours at room temperature. Primary antibody binding was visualized by probing the sections with an AlexaFluor555-conjugated goat anti-mouse antibody (Invitrogen, Cat. No. A21422) at a 1:1000 dilution in 20% DMSO/2% Triton X-100/2.5% BSA. The tissue sections were sealed into pouches as before and incubated for 2 days at 4 degrees with gentle agitation. After the secondary antibody incubation period, the slices were removed and washed as before and stored in the dark at 4 degrees C until imaging. A DAPI counterstain was added to the slices one hour prior to imaging.

CASR confocal immunofluorescence was performed using a three phase detection method with no antigen retrieval step. 5 micron thick FFPE sections were prepared using standard methods and blocked with Endogenous Avidin/Biotin Blocking Kit reagents (Invitrogen, Cat. No. 00–4303) following the manufacturer’s instructions. Following a brief additional blocking step in Cas-Block (Thermo-Fisher, Cat. No. 00–8120) the primary anti-CASR antibody (Novus, Cat. No. NB-120-19347, Clone 5C10, ADD) was added to a final concentration of 2 micrograms/ml and incubated for four hours at room temperature. After washing, a biotinylated anti-mouse antibody (Vector Labs, Cat. No. BA-9200) was added at a 1:1000 dilution and incubated for 30 minutes at room temperature. The slides were washed again, and then avidin-conjugated AlexaFluor488 (Life Technologies, Cat. No. A21370) was added to detect anti-CASR reactivity. The plasma membrane marker WGA conjugated to AlexaFluor 594 (Life Technologies, Cat. No. W11262) was added to visualize the plasma membrane. Finally, DAPI (Life Technologies, Cat. No. D1306) was added to stain nuclei. After washing, the slides were blotted dry and coverslipped in VectaMount media (Vector Labs, Cat. No. H-1000).

### Image analysis

Live-cell image stacks and static immunofluorescence images with their associated metadata information were exported as Zen software *lsm* files and analyzed using Fiji, an open-source ImageJ-based processing package (http://fiji.sc). For quantitation of Fluo-4 live-cell fluorescent intensity, the image stacks were converted into z-projections and overlays were created to align the Hoechst-stained nuclei with the Fluo-4 channel output. The Cell Counter module of ImageJ was employed to determine the total number of nuclei in each image field as a readout of cell number. Fluo-4 threshold fluorescence intensity was adjusted with the 3D Object Counter 2.0 tool using the 6 consecutive pre-stimulation images as a baseline reference for each tissue slice. Centroid maps were then created, excluding edge objects and using a minimal size filter of 5 microns. Corrected total cell fluorescent intensities (CTCF) were calculated by subtracting field background from individual object Integrated Density (IntDen) values for each cell using the formula CTCF = IntDen–(area of selected cell x mean field background intensity). Individual kinetic profiles of calcium responsiveness were generated by plotting fluorescence intensity as a function of time in seconds, as previously described [13]. The proportion of maximally responsive cells was calculated by dividing the number of cells exhibiting a rapid and sustained flux profile [13] by the total number of cells in the field (S14 Fig). The calcium EC50 metric was calculated by plotting the proportion of maximally responsive cells as a function of log calcium concentration.

For static image analysis, anti-PTH signal intensity was scored from z-projection overlays of the nuclear and AlexaFluor555 output channels. Intracellular PTH was scored by object mapping, using isotype-matched IgG-probed images to establish background threshold values. IntDen values within each demarcated cellular region were generated and adjusted for field background. Conditional intensity (fold change) was calculated by comparing field-median IntDen values under high or low calcium concentration.

Alignment of PTH immunofluorescence and flux activity was performed using the Coloc2 module of ImageJ. Images of live-cell flux activity at baseline and at 2 minutes after calcium stimulation were aligned with PTH immunofluorescence images of the same field in tissue slices fixed immediately after flux image capture. The nuclear staining channel in both the live-cell and fixed cell image fields were aligned using the registration plug-in tool of ImageJ to ensure precise superimposition prior to colocalization analysis. The Costes thresholding method [31, 32] set for 10 randomizations was then employed to detect colocalization of PTH immunoreactivity within flux-positive cells. Pearson’s correlation coefficients were calculated from matched image pairs representing at least two different z-axis planar depths in each field of view.

## Supporting Information

S1 Fig Variable patterns of CASR subcellular localization in parathyroid tumors. Immunofluorescence images of (A) normal parathyroid or (B) five different parathyroid adenoma specimens. Green = CASR. Blue = DAPI. Red = WGA. Normal parathyroid image is 400X. Parathyroid adenoma images are 200X.
(EPS)

S2 Fig Time-ordered image stacks of parathyroid tumor section response to ionomycin stimulation. Sequential images of Fluo-4-AM fluorescence (in green) and corresponding nuclear fields (Hoechst 33342 fluorescence, in blue) are provided in *lsm* format.
(DOCX)

S3 Fig Time ordered image stacks of parathyroid tumor section response to 2 mM calcium stimulation. Sequential images of Fluo-4-AM fluorescence (in green) and corresponding nuclear fields (Hoechst 33342 fluorescence, in blue) are provided in *lsm* format.
(ZIP)

S4 Fig Overlay of propidium iodide staining, a marker of non-viable cells, with intracellular flux response to 10 micromolar ionomycin, 10 nM thapsigargin, or 5 micromolar A23187. Propidium iodide = red. Fluo-4-AM = green.
(EPS)

S5 Fig Hoechst 33342 fluorescence image of nuclear staining. (TIF)

S6 Fig Enumerated nuclei identified using the Cell Counter module of ImageJ. (TIF)

S7 Fig Time-ordered image stacks of parathyroid tumor section response to 0.5 mM calcium. (ZIP)

S8 Fig Time-ordered image stacks of parathyroid tumor section response to 0.75 mM calcium. (TIF)

S9 Fig Time-ordered image stacks of parathyroid tumor section response to 1.0 mM calcium. (TIF)

S10 Fig Time-ordered image stacks of parathyroid tumor section response to 1.25 mM calcium. (TIF)

S11 Fig Time-ordered image stacks of parathyroid tumor section response to 3 mM calcium. (TIF)

S12 Fig Time-ordered image stacks of parathyroid tumor section response to 5 mM calcium. (TIF)

S13 Fig Time-ordered image stacks of parathyroid tumor section response to 10 mM calcium. (ZIP)

S14 Fig Proportion of cells exhibiting maximal response as a function of calcium concentration. (DOCX)

S15 Fig Characteristics of the patients whose tumors were analyzed in Fig 10. (DOCX)
