# Supplementary material for: A Novel Ex Vivo Method for Visualizing Live-Cell Calcium Response Behavior in Intact Human Tumors
Source: PLoS One. 2016 Aug 18;11(8):e0161134. doi: 10.1371/journal.pone.0161134 (PMC4990350; doi:10.1371/journal.pone.0161134)
Supplement: S2 Fig — Sequential images of Fluo-4-AM fluorescence (in green) and corresponding nuclear fields (Hoechst 33342 fluorescence, in blue) are provided in lsm format. (DOCX) [file pone.0161134.s002.docx]

Supplementary Data file S2 can be accessed through the following link to the Open Science Framework data repository:

https://urldefense.proofpoint.com/v2/url?u=https-3A__osf.io_cagj3_-3Fview-5Fonly-3Db1483caee4ae4a35a0ad52225ce9ea72&d=CwIGaQ&c=imBPVzF25OnBgGmVOlcsiEgHoG1i6YHLR0Sj_gZ4adc&r=saOksV0LJTYM5hHyvKRfwsqcrFolJPgLt0fhtuIyypE&m=pyoPfH9pnmhg0fGyEc_Io5moaj3Jk_C1cKbJINmTX_o&s=l3nGBpm7fHrXvRiSQKV7ysZ6wYiZ3ah
