# Supplementary figures and images for: A Novel Ex Vivo Method for Visualizing Live-Cell Calcium Response Behavior in Intact Human Tumors
Source: PLoS One. 2016 Aug 18;11(8):e0161134. doi: 10.1371/journal.pone.0161134 (PMC4990350; doi:10.1371/journal.pone.0161134)

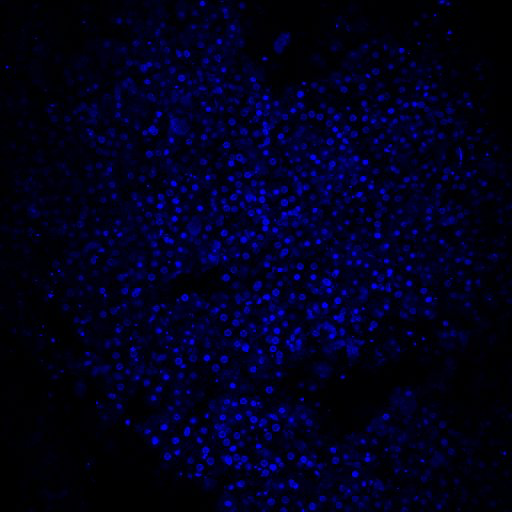

Supplement: S5 Fig — (TIF) [file pone.0161134.s005.tif]

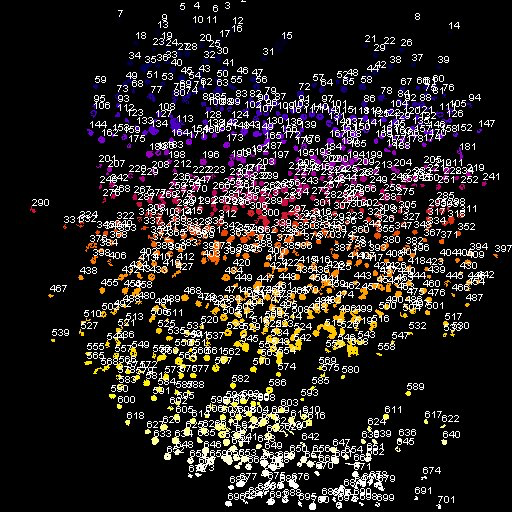

Supplement: S6 Fig — (TIF) [file pone.0161134.s006.tif]
