# Supplementary material for: A Novel Ex Vivo Method for Visualizing Live-Cell Calcium Response Behavior in Intact Human Tumors
Source: PLoS One. 2016 Aug 18;11(8):e0161134. doi: 10.1371/journal.pone.0161134 (PMC4990350; doi:10.1371/journal.pone.0161134)
Supplement: S14 Fig — (DOCX) [file pone.0161134.s014.docx]

| **Ca++ Conc. (mM)** | **n** | **# Max. Responders** | **% Max Response** |
| --- | --- | --- | --- |
| 0.5 | 169 | 0 | 0.0 |
| 0.75 | 527 | 17 | 3.2 |
| 1 | 392 | 11 | 2.8 |
| 1.25 | 497 | 9 | 1.8 |
| 2 | 250 | 34 | 13.6 |
| 3 | 403 | 80 | 19.9 |
| 5 | 380 | 168 | 44.2 |
| 10 | 381 | 174 | 45.7 |

**Table S12.** Calcium response distribution as a function of calcium concentration.
