# Supplementary material for: A Novel Ex Vivo Method for Visualizing Live-Cell Calcium Response Behavior in Intact Human Tumors
Source: PLoS One. 2016 Aug 18;11(8):e0161134. doi: 10.1371/journal.pone.0161134 (PMC4990350; doi:10.1371/journal.pone.0161134)
Supplement: S15 Fig — (DOCX) [file pone.0161134.s015.docx]

Supplementary Data Table S14

|  | **Patient 1** | **Patient 2** | **Patient 3** |
| --- | --- | --- | --- |
| Age | 55 | 65 | 75 |
| Gender | Female | Female | Female |
| Serum calcium (mg/dL) | 9.9 | 10.5 | 11.2 |
| iPTH (pg/ml) at diagnosis | 251 | 51 | 188 |
| Intraoperative PTH, start | 185 | 96 | 134 |
| Intraoperative PTH, post-resection | 34 | 38 | 22 |
| Severity indicators | Nephrolithiasis | Osteoporosis | None |
